# Whether Out-of-Bed Activity Restriction in the Early Postoperative Period of PELD Is Beneficial to Therapeutic Efficacy or Reduce Recurrence

**DOI:** 10.3389/fsurg.2022.860140

**Published:** 2022-05-09

**Authors:** Xiao Liang, Yexin Wang, Yaosheng Yue, Yanpeng Li, Chunyang Meng

**Affiliations:** ^1^Affiliated Hospital of Jining Medical University, Jining, China; ^2^Jiaxiang Peaple's Hospital, Jining, China

**Keywords:** PELD, out-of-bed activity, recurrence, pain, LDH

## Abstract

**Objective:**

To detect the influences of postoperative out-of-bed activity restriction on recurrence rate, low back and leg pain, functional rehabilitation after percutaneous endoscopic lumbar discectomy (PELD).

**Methods:**

In this research, 213 patients with lumbar intervertebral disc herniation (LDH) who underwent PELD were divided into the out-of-bed activity restriction group and out-of-bed activity non-restriction group. The visual analog scale (VAS) and Oswestry disability index (ODI) scores were used to evaluate postoperative clinical efficacy at 1 and 3 months after the operation, and to count the recurrence rates. All of these operations were performed between August 2017 and July 2020, and they were followed in the outpatient department for 12 months at least.

**Results:**

Both of the groups showed significantly lower VAS and higher ODI scores at 1 month and 3 months post-operation, respectively, when compared with pre-operation. At 1 month after the operation, the restriction group performed lower VAS scores of low back pain compared with the non-restriction group, but this advantage disappeared at 3months post-operation. However, there was no statistical difference in the VAS scores of leg pain and ODI scores between the two groups, neither at 1 nor 3 months after the surgery. The recurrence rate is significantly lower in the restriction group than in the non-restriction group at a 12-month follow-up after the surgery.

**Conclusion:**

Out-of-bed activity restriction in the early postoperative period of PELD could reduce LDH recurrence effectively, and it may relieve the low back pain to some extent. It has no benefit in the recovery of leg pain and functional rehabilitation.

## Introduction

With the development of endoscope instruments and minimally invasive techniques, percutaneous endoscopic lumbar discectomy (PELD) has been widely used in the treatment of lumbar intervertebral disc herniation (LDH). This kind of technique became more and more popular mainly attributed to its minimally invasive, early ambulant, fast rehabilitation, and short hospital stay (1, 2). Numerous studies (3–5) have shown that correct low back muscle exercise after surgery is crucial for functional recovery. On the other hand, it was observed that engaging in daily activities immediately after the surgery may lead to a higher recurrence rate, longer duration of pain, and even affect the therapeutic efficacy (6). So in the early stage after PELD, the choice of participating in daily activities right away or restricting out-of-bed activity remains to be discussed. In this study, we aimed to compare the effects of these two approaches for patients after the surgery, to explore which way may maximize the benefit for patients with LDH.

## Materials and Methods

### Patients

The study was approved by our institutional review board. All patients with LDH performed PELD between August 2017 to July 2020 at the Spine Surgery Department of Affiliated Hospital of Jining Medical College in Shandong, China. They were followed up in the outpatient department for 12 months at least.

#### Inclusion Criteria

(1) unilateral lower limb pain with or without low back pain, (2) single-level LDH, (3) failure of conservative treatment for more than 6 weeks, (4) nerve root compressed by herniated disc fragment which was confirmed by CT or/and MRI, and (5) postoperative radiographs verified that the herniated disc was completely removed.

#### Exclusion Criteria

(1) cauda equina syndrome, (2) recurrent LDH, (3) complicated with lumbar spinal stenosis, lumbar instability or spondylolisthesis, (4) lumbar spinal infection, tumor, deformity, (5) combined with other systemic diseases who cannot tolerate or cooperate surgery, (6) unable to follow up on schedule, and (7) postoperative radiographs verified that the herniated disc was not completely removed.

### General Information

All patients in this study were fully counted for the following indicators: gender, age, body mass index (BMI), operative level, modic change rate (confirmed by MRI), nucleus pulposus prolapse rate, operation time, intraoperative hemorrhage volume, hospital stay, and complication (epidural hematoma, nerve root injury, dural sac laceration, infection).

### Grouping

The out-of-bed activity restriction group demands that: in the first 2 weeks after surgery, out-of-bed activity time was limited to no more than 1 h each day, and no more than half an hour each time. Waist support must be guaranteed and a good habit of sitting up sideways must be established when getting out of bed. Furthermore, the waist bending, rotation, burden, and sedentariness were not permitted until 2 weeks post-operation. During bed stay, the patient performed a five-point support exercise to strengthen lower back muscles, ankle pump movement to prevent thrombosis and muscle atrophy, straight leg lifting exercise to prevent nerve root adhesion after postoperation.

The out-of-bed activity non-restriction group demands that: the patients start to walk with waist support and participate in daily activities the day after surgery. The intensity of labor was based on their own condition, but the weight-bearing and waist activity should be avoided.

Two kinds of rehabilitation programs were provided to the postoperative patients of PELD, a total of 213 patients chose the program completely depending on their own wishes. There were 108 patients in the out-of-bed activity restriction group and 105 patients in the out-of-bed activity non-restriction group.

### Surgical Procedure

According to the patients' own condition of LDH, they performed PELD successfully with two endoscopic approaches: the transforaminal (TF) approach and the interlaminar (IL) approach.

**Figure 1:**
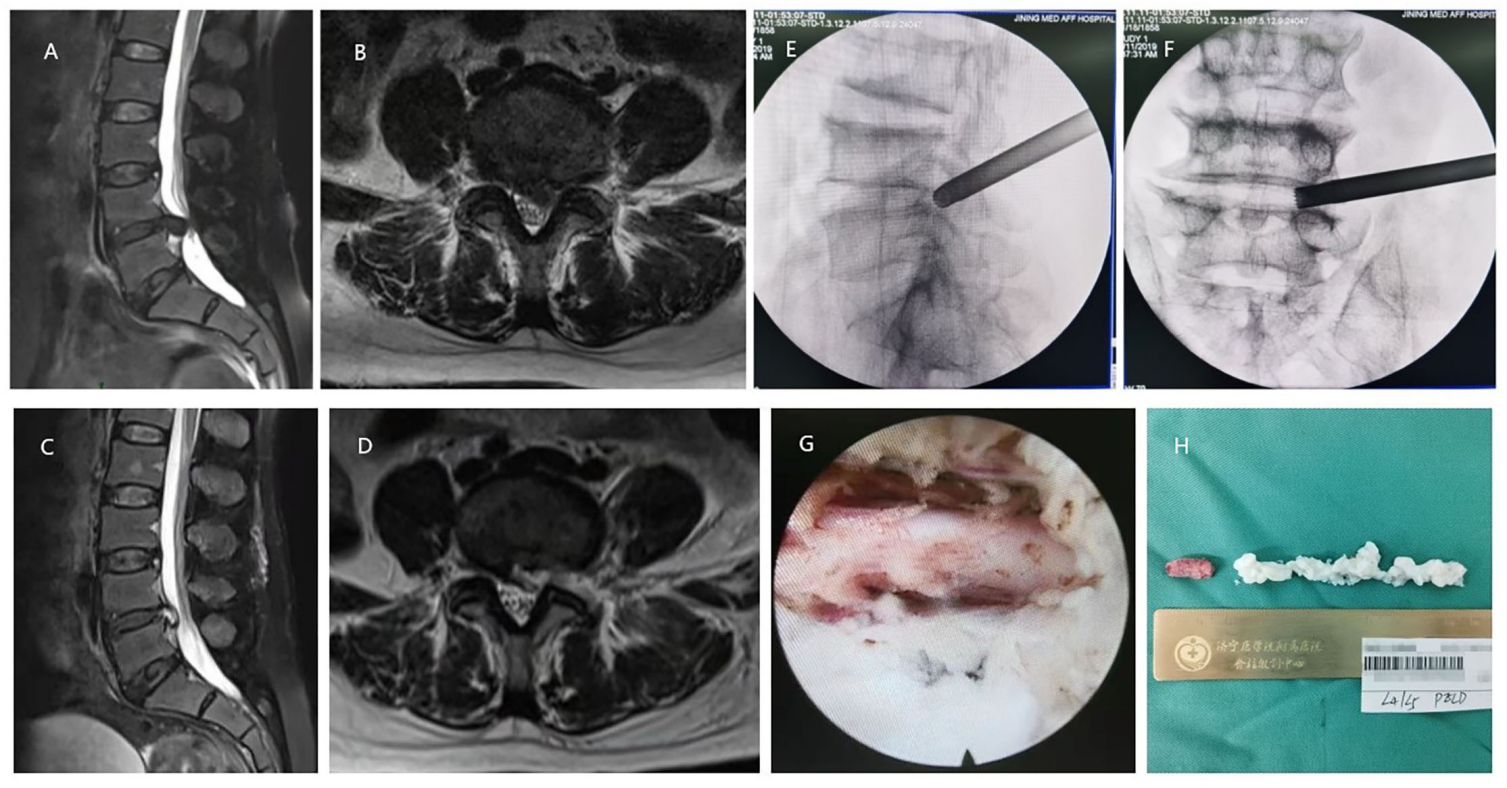
A 67-year-old female with the L4/5 lumbar intervertebral disc herniation (LDH) underwent percutaneous endoscopic lumbar discectomy (PELD) with the approach of transforaminal. **(A,B)** Preoperative MRI showed LDH was located at the L4/5 level. **(C,D)** Postoperative MRI revealed the herniated nucleus pulposus tissues were removed and the nerve root was decompressed. **(E)** Intraoperative C-arm fluoroscopy showed the location of the working channel on lateral film. **(F)** Intraoperative C-arm fluoroscopy showed the location of the working channel on anteroposterior film. **(G)** The decompressed nerve root under endoscopic view. **(H)** Resected nucleus pulposus tissues in the operation.

Transforaminal-PELD was performed under local anesthesia, patients were placed in the prone position on a radiolucent table and the puncture sites for the TF approach were marked under C-arm fluoroscopy. The final target point of the puncture needle was at the posterosuperior of the vertebra inferior on the lateral image and at the medial pedicular line on the anteroposterior image. A solution of 0.5% lidocaine and 0.25% ropivacaine is injected for infiltration anesthesia. An incision of nearly 7 mm was made at the puncture point of the skin, then the guidewire was put through the puncture needle, and a series of expansion channels were sequentially inserted along the guidewire to dilate the surgical channel. The circular saw was used to remove the tip and ventral part of the superior articular, to expand the intervertebral foramen and spine canal adequately. After the endoscopic insertion into the surgical area through the cannula, radiofrequency ablation was used to stop the bleeding and to expose the nerve root compressed by the herniated disc tissues. Finally, the herniated fragment was removed by nucleus pulposus forceps, and the synechia was separated by radiofrequency ablation, so as to loosen the nerve root and dural sac (Figure 1).

**Figure 2:**
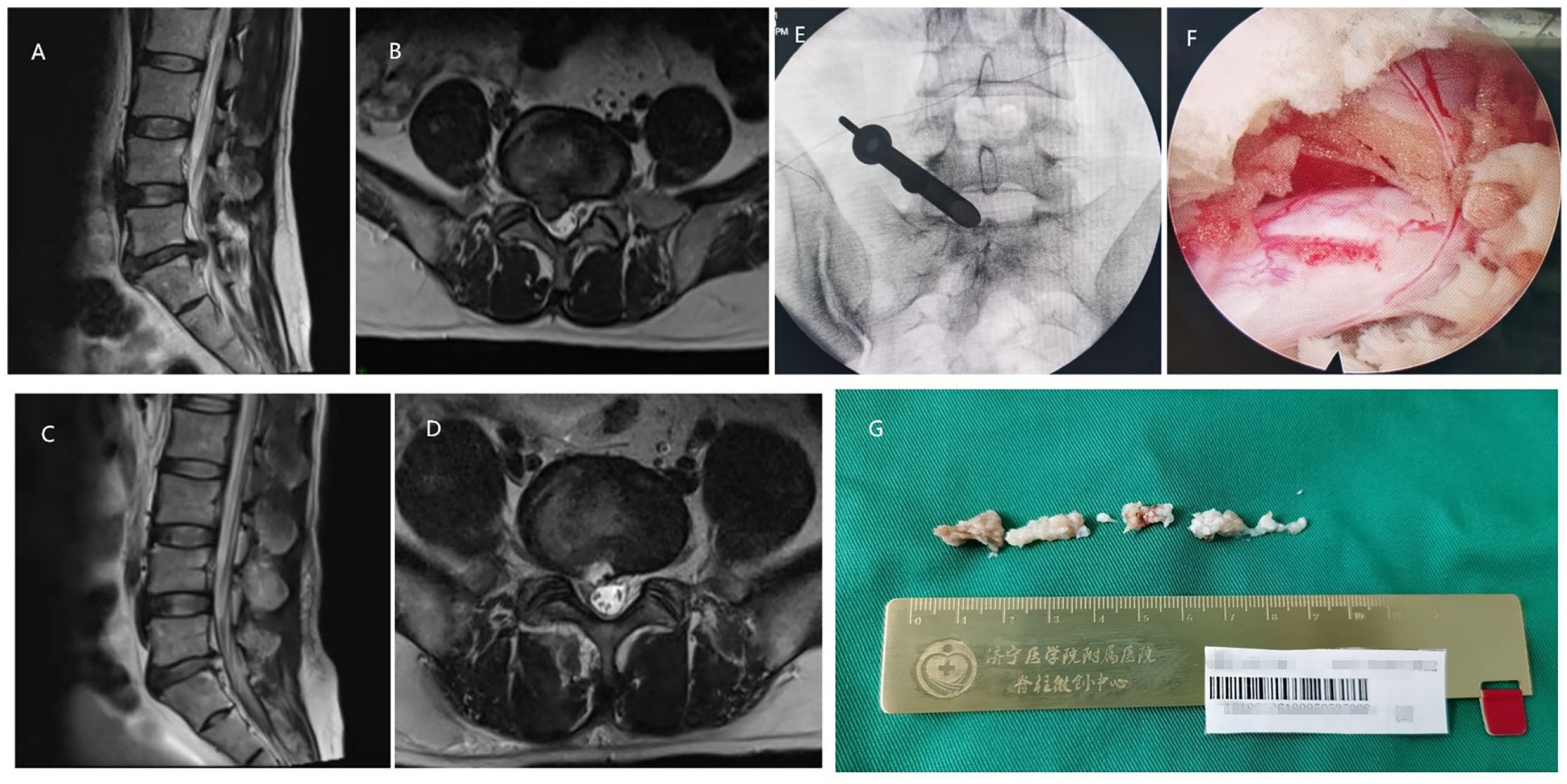
A 56-year-old male with the L5/S1 LDH underwent PELD with the approach of interlaminar. **(A,B)** Preoperative MRI showed LDH was located at the L5/S1 level. **(C,D)** Postoperative MRI revealed the herniated nucleus pulposus tissues were removed and the nerve root was decompressed. **(E)** Intraoperative C-arm fluoroscopy showed the location of the working channel. **(F)** The decompressed nerve root under endoscopic view. **(G)** Resected nucleus pulposus tissues in the operation.

Interlaminar-PELD was performed under general anesthesia, and the patients were placed in the prone position on a radiolucent table. With the help of C-arm fluoroscopy, the entry point was marked on the affected side of the back skin 0.5 cm next to the spinous process line. And the exact location was called the V-point, which is the intersection of the inferior margin of the vertebral plate superior and the superior margin of the vertebral plate inferior on the anteroposterior image. An incision of nearly 7 mm was made at the entry point of the skin, and a series of expansion channels were sequentially inserted into the surface of the ligamentum flavum. Then, the ligamentum flavum and soft tissue around it were removed by nucleus pulposus forceps and scissors under endoscopic observation until the spinal canal was revealed. After the dural sac and nerve root were completely exposed, the tongue of the working cannula was inserted and rotated into the lateral nerve root. Released the adhesion around the nerve with the use of radiofrequency bipolar, and push the nerve root softly to the direction of centerline, to protect the nerve and expose the prominent nucleus pulposus tissue. Removed the prominent nucleus pulposus by various nucleus pulposus forceps. Finally, examined the remaining herniated fragment and the bleeding points in the spinal canal, then checked the flexibility of the nerve root once again (Figure 2).

### Evaluation Methods

**Figure 3:**
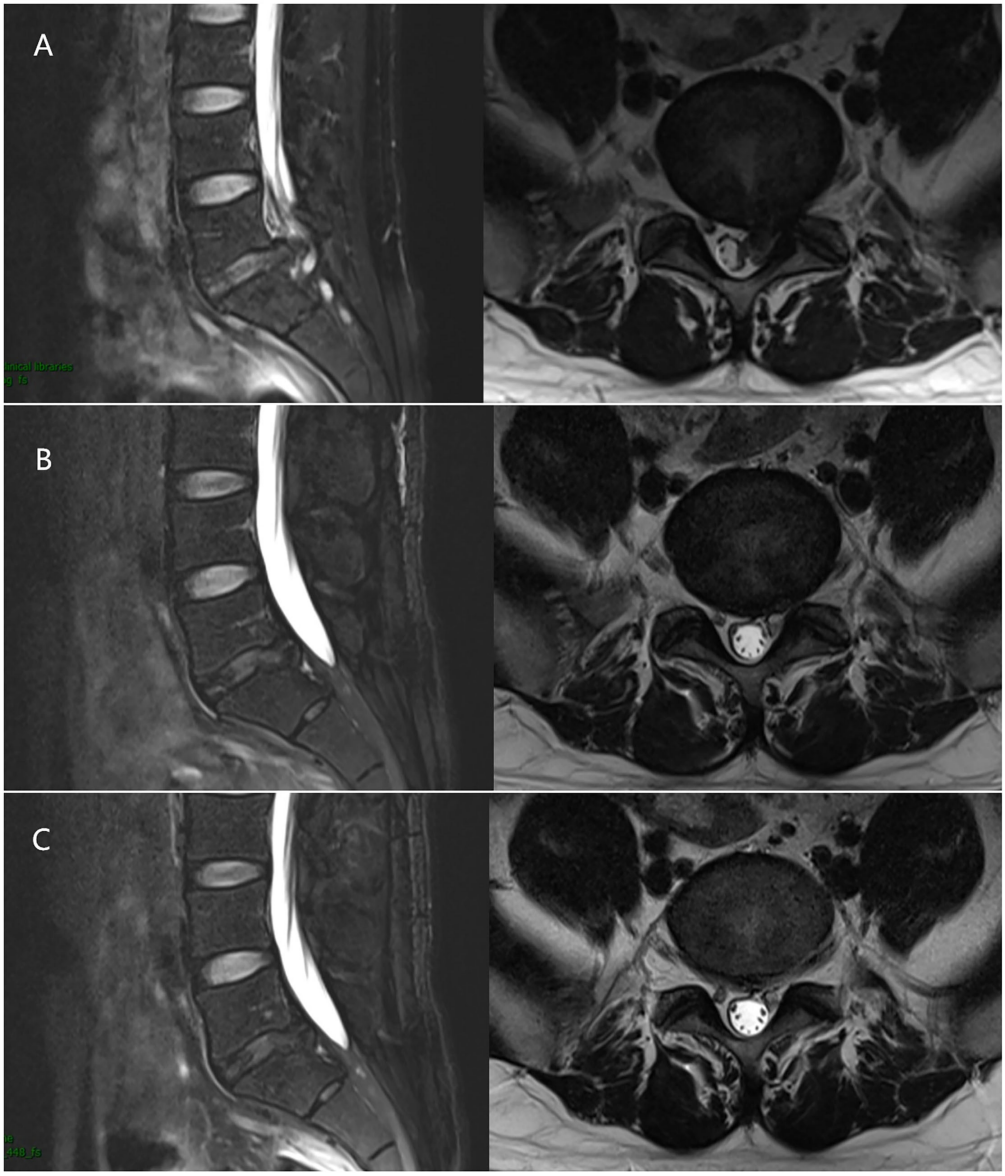
A 33-year-old male with the L5/S1 LDH underwent PELD. **(A)** Preoperative MRI showed LDH was located at the L5/S1 level. **(B)** 1 month after surgery, MRI revealed the nerve root was decompressed without recurrence. **(C)** 3 months after surgery, MRI revealed the nerve root was decompressed without recurrence as well.

Low back pain and lower limb pain were evaluated respectively, by the visual analog scale (VAS), 1 month postoperatively and 3 months postoperatively. The patient's functional disorder conditions were evaluated by Oswestry disability index (ODI) scores at the above time points. An imaging review was performed during pain assessment (Figure 3). All patients were followed up in the outpatient department for 12 months at least, and the recurrences were recorded.

### Statistical Analysis

The statistical data were analyzed by the SPSS 23.0 software (IBM, NY, USA). Quantitative data were expressed as mean ± SD. The paired *t*-test was used to compare the difference of continuous variables between the two groups. The chi-squared test was used to compare the difference of dichotomous variables between the two groups. *P* < 0.05 was considered to be statistically significant.

## Results

### Demographic Characteristics

There was no significant difference between the two groups in gender, age, BMI, operative level, modic change rate, nucleus pulposus prolapse rate, operation time, intraoperative hemorrhage volume, hospital stay, and complication (Table 1).

**Table 1:** Demographic characteristics.

	**Restriction group**	**Non-restriction group**	***P*-value**
Number of patients	108	105	
Gender male: (%)	59 (54.63%)	56 (53.33%)	0.85
Age (years)	51.45 ± 12.70	49.56 ± 13.96	0.30
BMI	26.14 ± 2.46	25.84 ± 2.46	0.37
Operative level:L3-4 (%)	5 (4.63%)	4 (3.80%)	1.00
Operative level:L4-5 (%)	56 (51.85%)	52 (49.52%)	0.73
Operative level:L5-S1 (%)	47 (43.52%)	49 (46.67%)	0.64
Modic change:n (%)	29 (26.85%)	39 (37.14%)	0.11
Nucleus pulposus prolapse:n (%)	68 (62.96%)	55 (52.38%)	0.12
Operation time (min)	84.42 ± 23.19	81.49 ± 23.94	0.37
Intraoperative hemorrhage volume (ml)	16.28 ± 6.37	17.72 ± 6.88	0.11
Hospital stay (days)	3.36 ± 0.93	3.33 ± 0.85	0.82
Complication: n (%)	7 (6.48%)	5 (4.76%)	0.59

### Comparison of VAS Scores

Compared with the VAS scores of low back and leg pain preoperatively, the scores at 1 and 3 months postoperatively were significantly declined in both of the 2 groups. At 1month after the operation, the restriction group performed lower VAS scores of low back pain compared with those in the non-restriction group, and the difference was statistically significant. However, the difference of VAS scores in low back pain between the 2 groups disappeared at 3 months postoperatively. There was no statistical difference in the VAS scores of leg pain between the two groups neither at 1 month nor 3months postoperatively (Table 2).

**Table 2:** Comparison of VAS scores.

**VAS scores**		**Restriction group**	**Non-restriction group**	***P*-value**
Low back pain	Preoperative	3.84 ± 1.25	3.91 ± 1.19	0.67
	1 month postoperative	1.14 ± 0.66a	1.60 ± 0.91a	0.00
	3 months postoperative	1.06 ± 0.75a	1.12 ± 0.80a	0.52
Leg pain	Preoperative	7.05 ± 1.38	7.25 ± 1.52	0.31
	1 month postoperative	1.99 ± 0.78a	1.88 ± 0.83a	0.30
	3 months postoperative	1.60 ± 0.84a	1.59 ± 0.87a	0.92

*a, Statistically significant difference compared with preoperative VAS scores*.

### Comparison of ODI Scores

In both of the two groups, the patients' ODI scores significantly improved at 1 and 3 months postoperatively compared with those preoperatively. But there were no statistically significant differences in ODI scores between the two groups at 1 and 3 months postoperatively (Table 3).

**Table 3:** Comparison of the ODI scores.

**ODI scores**	**Restriction group**	**Non-restriction group**	***P*-value**
Preoperative	53.01 ± 15.50	53.43 ± 14.65	0.84
1 month postoperative	12.56 ± 7.36b	13.60 ± 7.71b	0.31
3 months postoperative	8.04 ± 3.75b	8.90 ± 3.99b	0.10

*b, Statistically significant difference compared with preoperative ODI scores*.

### Comparison of Recurrence Rate

All the patients were followed up for 12 months after the operation, and the recurrence cases were confirmed by clinical symptoms and image logical examinations 0.5 cases of recurrence were revealed in the restriction group, whose recurrence rate was 4.63%, and all of them were underwent operation again. Correspondingly, 13 cases of recurrence were revealed in the non-restriction group, which recurrence rate was 12.38%. In total, 10 of them underwent operation again, and the rest recovered with conservative treatment. The restriction group showed a significantly lower recurrence rate than that in the non-restriction group with statistical differences (Table 4).

**Table 4:** Comparison of recurrence rate.

	**Restriction group**	**Non-restriction group**	***P*-value**
Recurrence (%)	5 (4.63%)	13 (12.38%)	0.042

## Discussion

With the development of endoscopic technology, PELD has gradually become the mainstream surgical method for the treatment of LDH. It is mainly divided into the TF and the IL different surgical approaches and is widely used in clinical practice. Even when compared with other types of minimally invasive surgery, such as MIS-TLIF and unilateral biportal endoscopic discectomy, PELD performed obviously less intraoperative blood loss, shorter operative time, lighter low back, and leg pain postoperative (7, 8). Although it has minimally invasive features, PELD requires the destruction of back soft tissue and lumbar disc structure, which inevitably leads to postoperative pain and recurrence in patients, and recurrence rates of LDH have been reported in the literature ranging from 5 to 15 percent (9). Numerous studies have shown the factors of recurrence as age, BMI, disc degeneration, surgical approaches, early ambulation, postoperative instability, or hypermobility (2, 6, 10).

Almost the vast majority of surgeons require patients to get out of bed within 1–3 days after surgery for early functional exercise. However, we found that early out-of-bed activity restriction significantly reduced low back pain in this research. Early participation in daily activities may increase the load on the lumbar spine and enhance intradiscal pressure (IDP). From the supine position to the standing position, the IDP can increase significantly, and the flexion position can increase the IDP further. However, the change in intradiscal pressure may lead to the nucleus pulposus tissue re-entering the spinal canal through the annulus fibrosus breach and induced clinical symptoms, which can be seen as the pathological basis of recurrent LDH.

The correlation between compression force and intervertebral disc degeneration has been confirmed by many mechanically induced disc degeneration studies (11, 12). In the experimental model of Guehring (13), prolonging the time of compression may lead to more severe disc degeneration. This conclusion reflects the result in our study that restricting out-of-bed activity time in the early postoperative period of PELD could reduce recurrence rates. Although PELD can effectively remove the nucleus pulposus tissues to achieve the satisfactory therapeutic effect, most annulus fibrosus defects remain unrepaired at last, which might affects intervertebral disc integrity and stability. From the perspective of biomechanics, Fujii (14) confirmed that fibrous ring injury significantly altered several biomechanical parameters, such as axial range of motion, torsional stiffness, torque range, neutral zone, and stress-relaxation compared to the intact intervertebral disc. As the intervertebral disc is repaired, some biomechanical parameters gradually recover, which indicates that the intervertebral disc defect encapsulation improved its stability to some extent. The repair of the intervertebral disc is usually limited to the annulus fibrosus outer layer. Results of an animal study revealed that, after pressure was removed from the rabbit, signs of intervertebral disks tissue recovery were observed on a biologic, cellular, and biomechanical level. Its manifest disc regeneration can be induced by axial dynamic distraction (15). So by limiting the time and intensity of the postoperative ambulation, on the one hand, the recurrence can be reduced by reducing the intervertebral disc pressure, on the other hand, by reducing the intervertebral disc axial stress, so as to improve tissue repair efficiency in the intervertebral disc, rebuild the outer fiber ring to restore the biomechanical stability as early as possible, which also can reduce the recurrence rate.

In fact, it can be found sometimes that patients with successfully completed PELD still suffer low back and lower limbs pain, which persists for a period of time, even though nucleus pulposus residue has been ruled out by imaging examination. Eliminating the factor of early recurrence, incomplete removal of the herniated disc, and nerve root injury, Zhang (16) found that 10.4% of patients had short-term rebound low back and leg pain usually began within 1 month after PELD, then the symptoms were relieved after conservative treatment. Research revealed that the nerve growth into the intervertebral disc through the fissures of the fibrous ring and express substance P plays an important role in the pathogenesis of chronic low back pain caused by the destruction of annulus fibrosus in surgery (17). Internal disc disruption is a pathologic condition that can result in discogenic pain (18). After surgery interference, intervertebral disc nucleus pulposus tissue may be mixed with vertebral endplate fragments, fibrous ring debris, liquid, or gas, and small fragments of the endplate and fibrous ring may fall into the intervertebral disc degeneration region. With the change of the position, an acute discogenic pain will be caused when the fragments are just in the main load-bearing area. Additionally, it is quite common to destroy parts of zygapophysial joints for dilating the foramen, but it causes joint instability to some extent, in fact, it will lead to instability (19). When the patients resume daily activities just out of surgery, a sudden load change on the joint may lead to discomfort. Consistent with the results of this study, even if there is no recurrence, excessive and premature postoperative activity is more likely to cause postoperative low back pain.

Some research found that there were 10.4 to 20.4% of patients reappeared lower limb pain after PELD (16, 20). This kind of reappeared lower limb pain is also very common in the cases we observed. That's probably due to intraoperative nerve pulling and stimulation, the nerve root is still in a state of inflammation and edema post-operation, even if decompression is sufficient (21). Furthermore, local hematoma formed after herniated intervertebral disc tissue is removed may take time to absorb, and insufficient blood supply to local vessels can further aggravate inflammatory edema. In this study, out-of-bed activity time restriction didn't benefit reducing lower limb pain or raising ODI scores. The possible explanation is that, in the absence of recurrence, out-of-bed activity did not change the anatomical structure of the spinal canal contents, resulting in no further effect on lower limbs pain. The restriction group reached the same level as the non-restriction group in functional recovery profited from rigorous rehabilitation exercise during bedtime. It seems to be that early activity time has little impact on lower limbs pain and recovery of motor function post-operation.

## Conclusion

This study detected that the lack of appropriate restrictions on out-of-bed activity time in the early period after PELD should be one risk factor for recurrence. It may affect the recovery of low back pain in the early postoperative period just like 1-month post-operation, however, its influence disappeared 2 months later. Out-of-bed activity time in the early period after PELD has no effect on the recovery of lower limbs pain or the ability to participate in daily activities.

## Data Availability Statement

The original contributions presented in the study are included in the article/supplementary material, further inquiries can be directed to the corresponding author.

## Ethics Statement

The studies involving human participants were reviewed and approved by Medical Ethics Committee of Affiliated Hospital of Jining Medical College, Jining Medical College. Written informed consent to participate in this study was provided by the participants' legal guardian/next of kin. Written informed consent was obtained from the individual(s) for the publication of any potentially identifiable images or data included in this article.

## Author Contributions

CM was responsible for the design of research and formulation of experimental methods, performed PELD surgery as the chief surgeon, and revised and finalized the article. XL participated in the screening and enrollment of the patients underwent PELD surgery for this research, recorded the general information and the operation data of patients, participated in the operation, and wrote this article. YW participated in the operations, educated the patient about out-of-bed activity restriction requirements postoperatively, was responsible for the follow-up of patients, and counted and recorded the patients who got recurrence after surgery. YY participated in data arrangement and statistical analysis. YL was responsible for the preoperative and postoperative VAS and ODI evaluation of patients. All authors contributed to the article and approved the submitted version.

## Funding

This study was supported by National Natural Science Foundation of China (No.81974345) and Natural Science Foundation of Shandong Province (No. ZR2019PH068).

## Conflict of Interest

The authors declare that the research was conducted in the absence of any commercial or financial relationships that could be construed as a potential conflict of interest.

## Publisher's Note

All claims expressed in this article are solely those of the authors and do not necessarily represent those of their affiliated organizations, or those of the publisher, the editors and the reviewers. Any product that may be evaluated in this article, or claim that may be made by its manufacturer, is not guaranteed or endorsed by the publisher.
